# Improving External Rotations in Psychiatry Residency Programs: A Mixed-Methods Analysis

**DOI:** 10.1192/j.eurpsy.2025.2374

**Published:** 2025-08-26

**Authors:** J. P. Carrasco, J. Esteve, C. Conde-Pumpido, B. Herraiz, J.-I. Etxeandia-Pradera, E. Aguilar

**Affiliations:** 1 Hospital Provincial Castellón, Castellón; 2 Hospital Clínico Universitario de Valencia, Valencia, Spain

## Abstract

**Introduction:**

External rotations allow psychiatry residents to gain exposure to different clinical settings and practices. However, the availability and quality of these rotations vary significantly across training units. This study evaluates residents’ experiences with external rotations and identifies areas for improvement.

**Objectives:**

To assess the time allocated for external rotations in different training units and explore residents’ suggestions for improving these rotations.

**Methods:**

A cross-sectional survey was distributed to psychiatry residents in Spain. Quantitative data regarding the total time allowed for external rotations was collected, and qualitative responses were analyzed to identify recurring themes for improvement.

**Results:**

A total of 109 responses were analyzed. Quantitatively, 60% of residents reported being allowed 1 to 3 months for external rotations, while 25% stated they had more than 3 months. Only 15% indicated that external rotations were not offered in their unit. Qualitative analysis revealed that the main areas for improvement included more flexibility in choosing rotation locations (40%), better financial support for rotations outside of the home institution (35%), and greater clarity in the application process for external rotations (25%).

**Table:** 

Rotation Time Allowed	Percentage (%)
1 to 3 months	60
More than 3 months	25
No external rotations offered	15

**Table:** 

Theme	Percentage (%)
Flexibility in location choice	40
Financial support	35
Clarity in application process	25

**Conclusions:**

The majority of psychiatry residents are allocated 1 to 3 months for external rotations, but many see room for improvement in terms of flexibility, financial support, and clarity in the application process. Enhancing these aspects could make external rotations more accessible and beneficial for residents.

**Disclosure of Interest:**

None Declared

